# Supplementary figures and images for: High efficient de novo root-to-shoot organogenesis in Citrus jambhiri Lush.: Gene expression, genetic stability and virus indexing
Source: PLoS One. 2021 Feb 19;16(2):e0246971. doi: 10.1371/journal.pone.0246971 (PMC7894961; doi:10.1371/journal.pone.0246971)

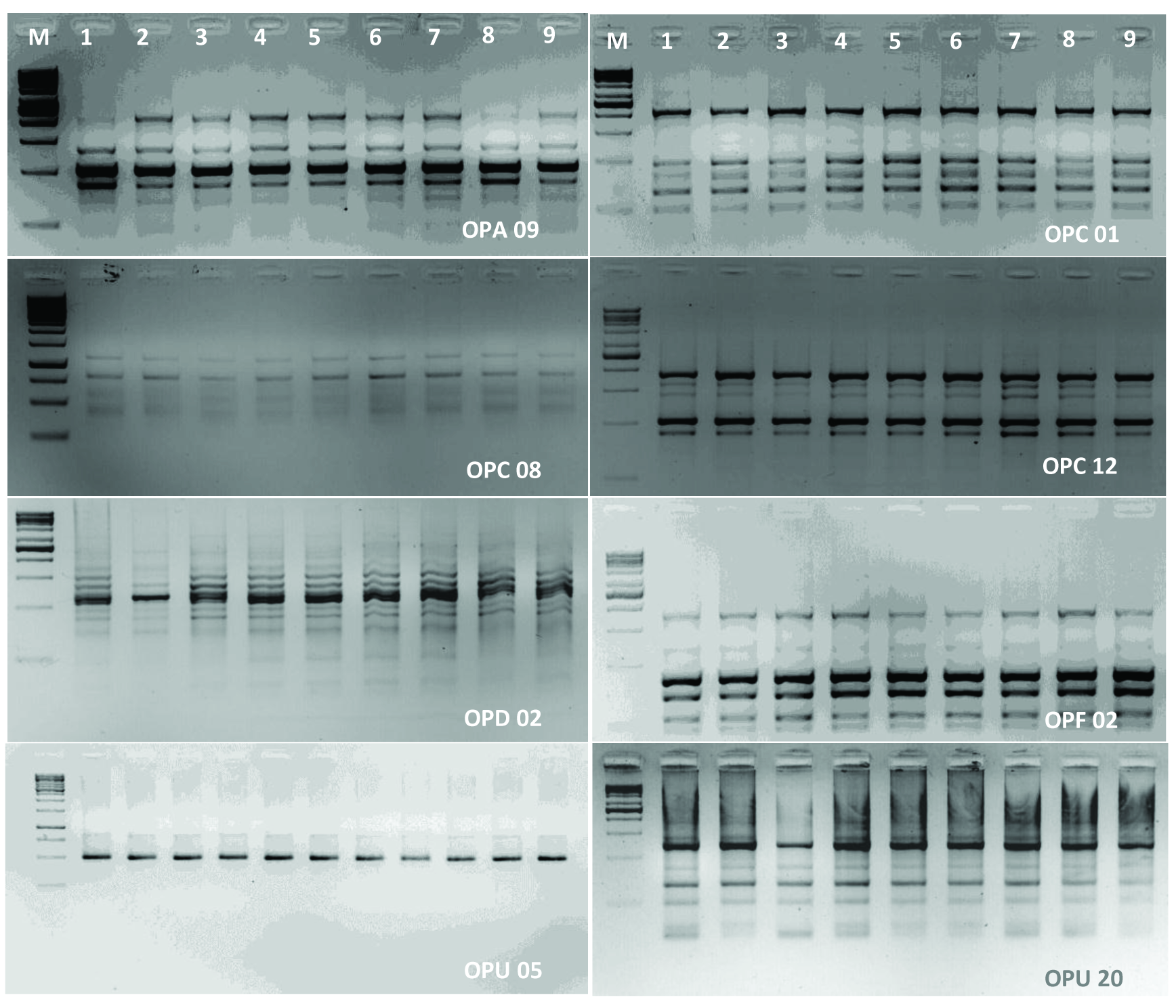

Supplement: S1 Fig — [Lane M: 1 kb DNA ladder for RAPD; Lane 1: Mother plant from Kachai village, Ukhrul, Manipur; Lane 2: In vitro seedlings; Lane 3: Seedlings planted at Langol farm of ICAR, Manipur; Lane 4: Seedlings planted at polyhouse of ICAR, Manipur; Lane 5: Regenerants obtained from MSN+BAP 1.0+GA3 1.0 mg L-1; Lane 6: Regenerants obtained from MSN+BAP 1.0+GA3 2.0 mg L-1; Lane 7: Plantlets obtained from ½MSN; Lane 8: Plantlets obtained from ½MSN+NAA 1.0 mg L-1; Lane 9: Plantlets obtained from ½MSN+IAA 1.0 mg L-1]. DOI 10.17605/OSF.IO/XWT8B. (TIF) [file pone.0246971.s001.tif]

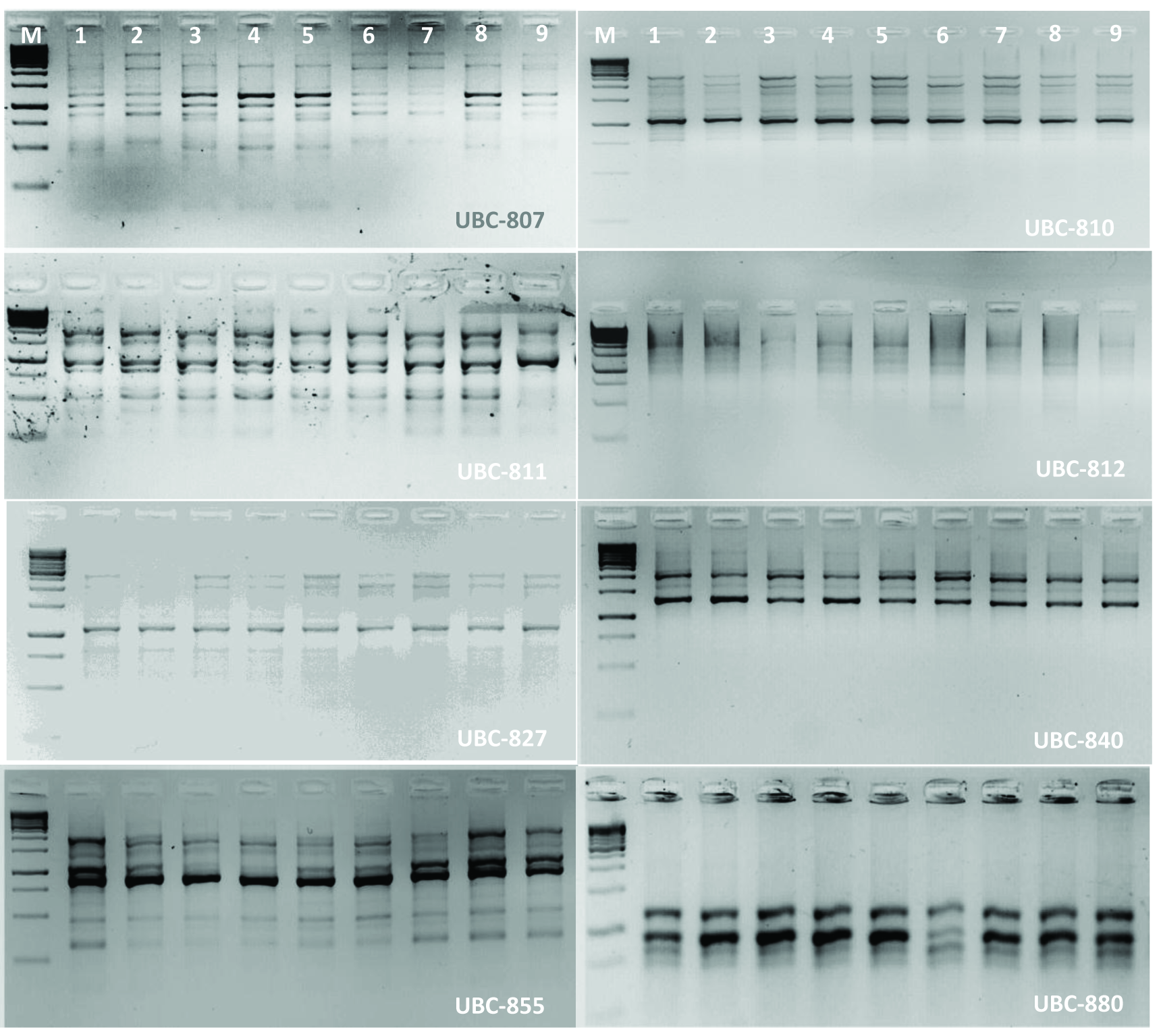

Supplement: S2 Fig — [Lane M: 1 kb DNA ladder for ISSR; Lane 1: Mother plant from Kachai village, Ukhrul, Manipur; Lane 2: In vitro seedlings; Lane 3: Seedlings planted at Langol farm of ICAR, Manipur; Lane 4: Seedlings planted at polyhouse of ICAR, Manipur; Lane 5: Regenerants obtained from MSN+BAP 1.0+GA3 1.0 mg L-1; Lane 6: Regenerants obtained from MSN+BAP 1.0+GA3 2.0 mg L-1; Lane 7: Plantlets obtained from ½MSN; Lane 8: Plantlets obtained from ½MSN+NAA 1.0 mg L-1; Lane 9: Plantlets obtained from ½MSN+IAA 1.0 mg L-1]. DOI 10.17605/OSF.IO/JBZUX. (TIF) [file pone.0246971.s002.tif]
