# Supplementary material for: High efficient de novo root-to-shoot organogenesis in Citrus jambhiri Lush.: Gene expression, genetic stability and virus indexing
Source: PLoS One. 2021 Feb 19;16(2):e0246971. doi: 10.1371/journal.pone.0246971 (PMC7894961; doi:10.1371/journal.pone.0246971)

RAW IMAGES-RAPD (OPA-09)

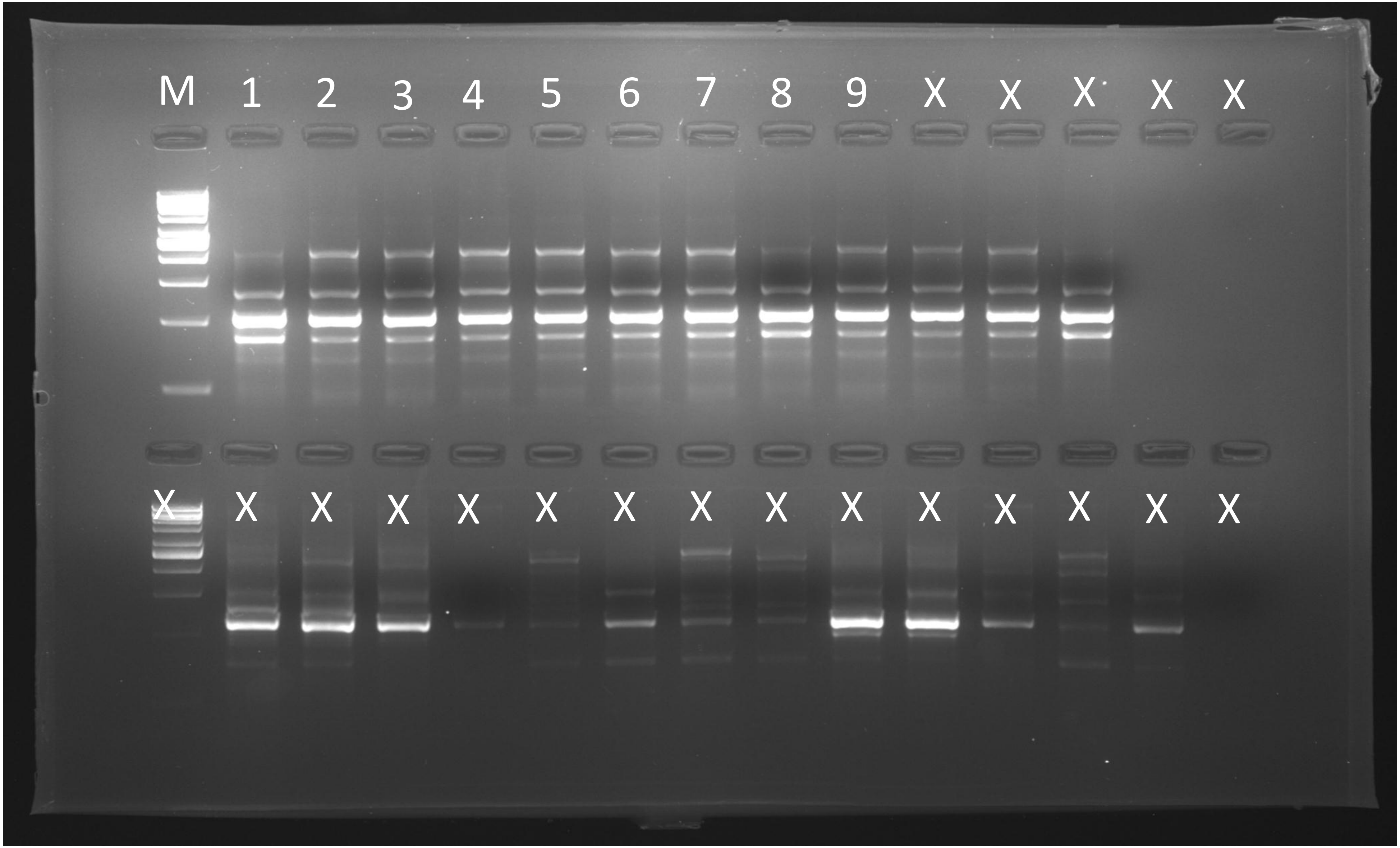

RAW IMAGES-RAPD (OPC-01)

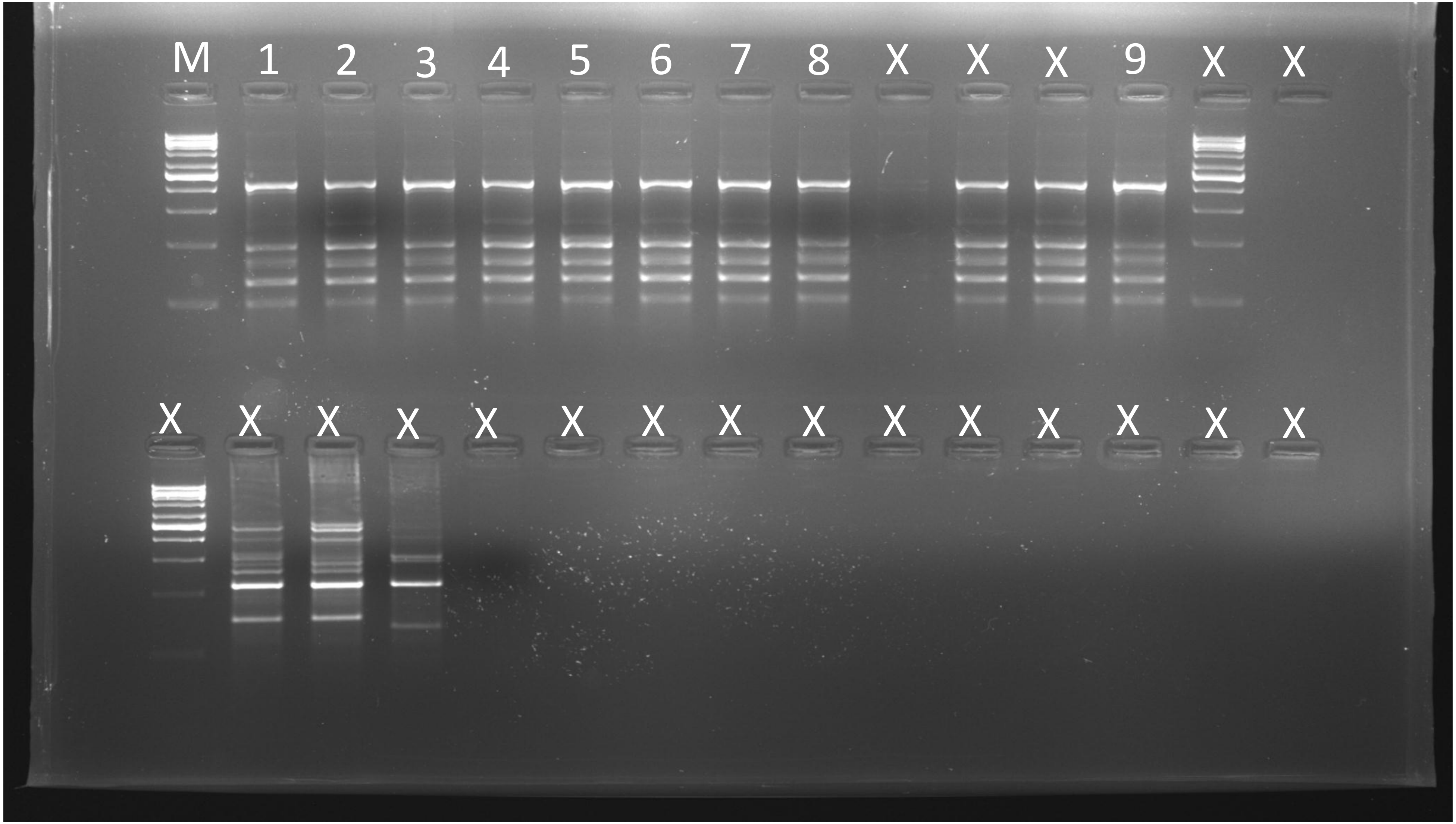

RAW IMAGES- RAPD (OPC-08)

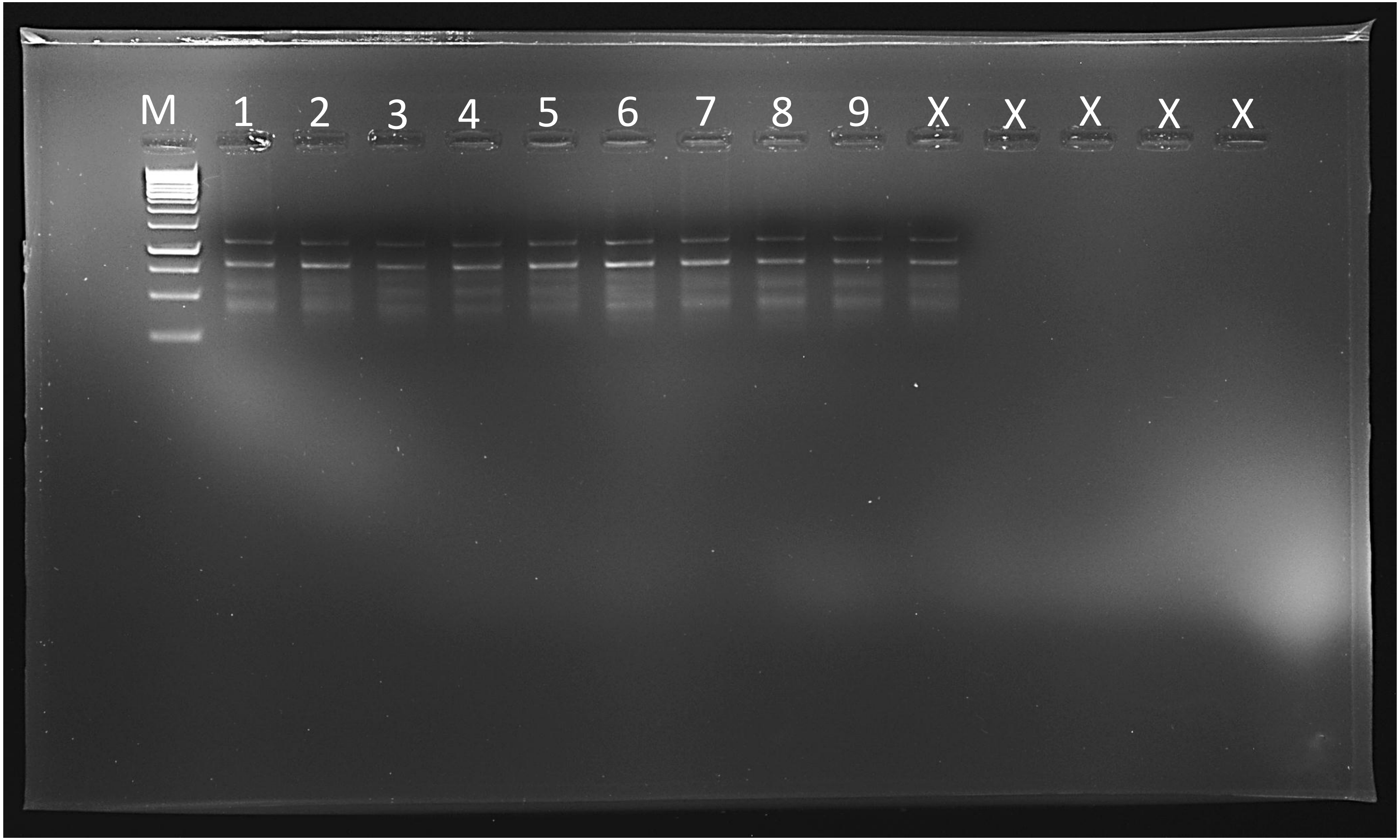

RAW IMAGES- RAPD (OPC-12)

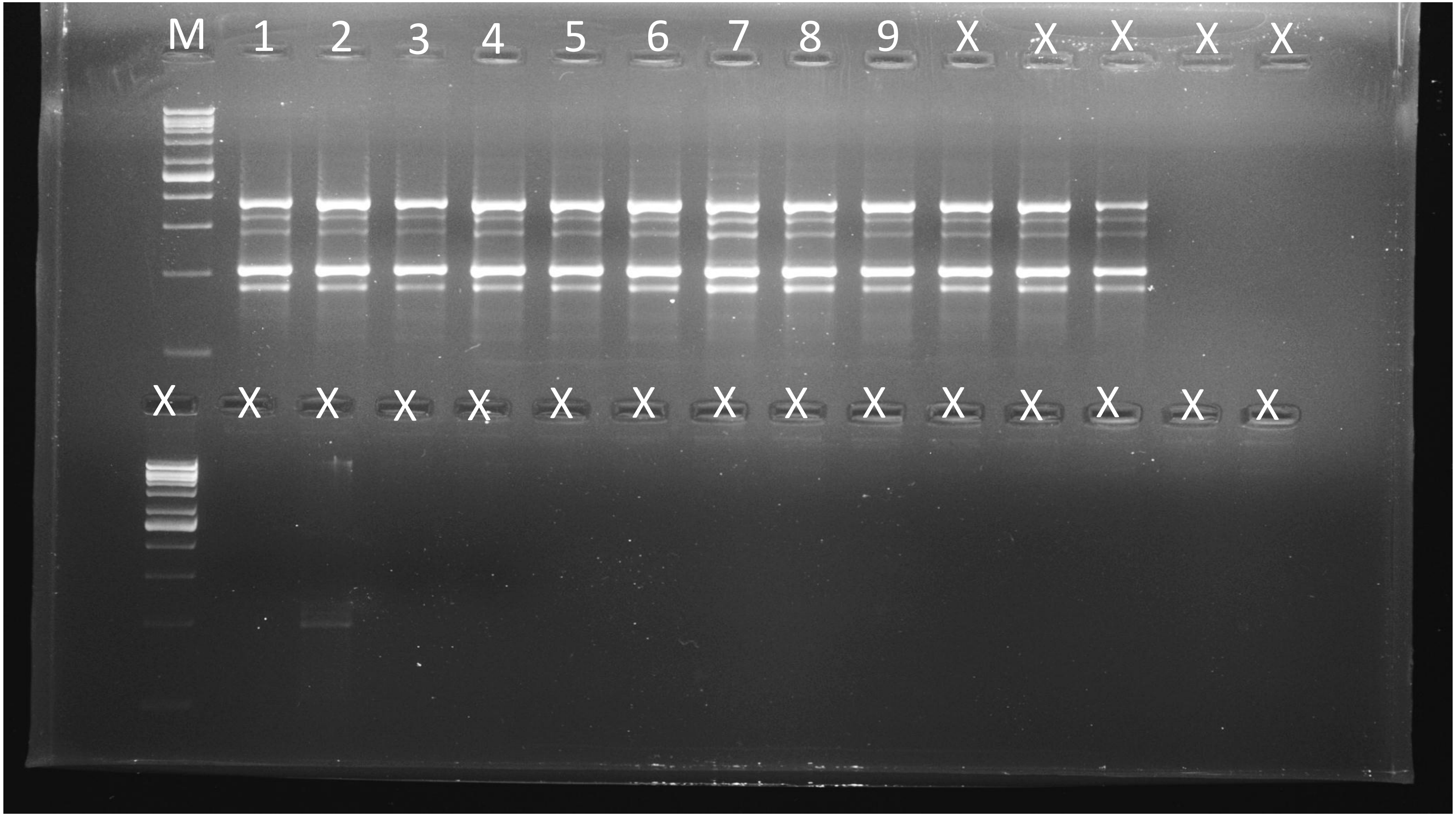

RAW IMAGES- RAPD (OPD-02)

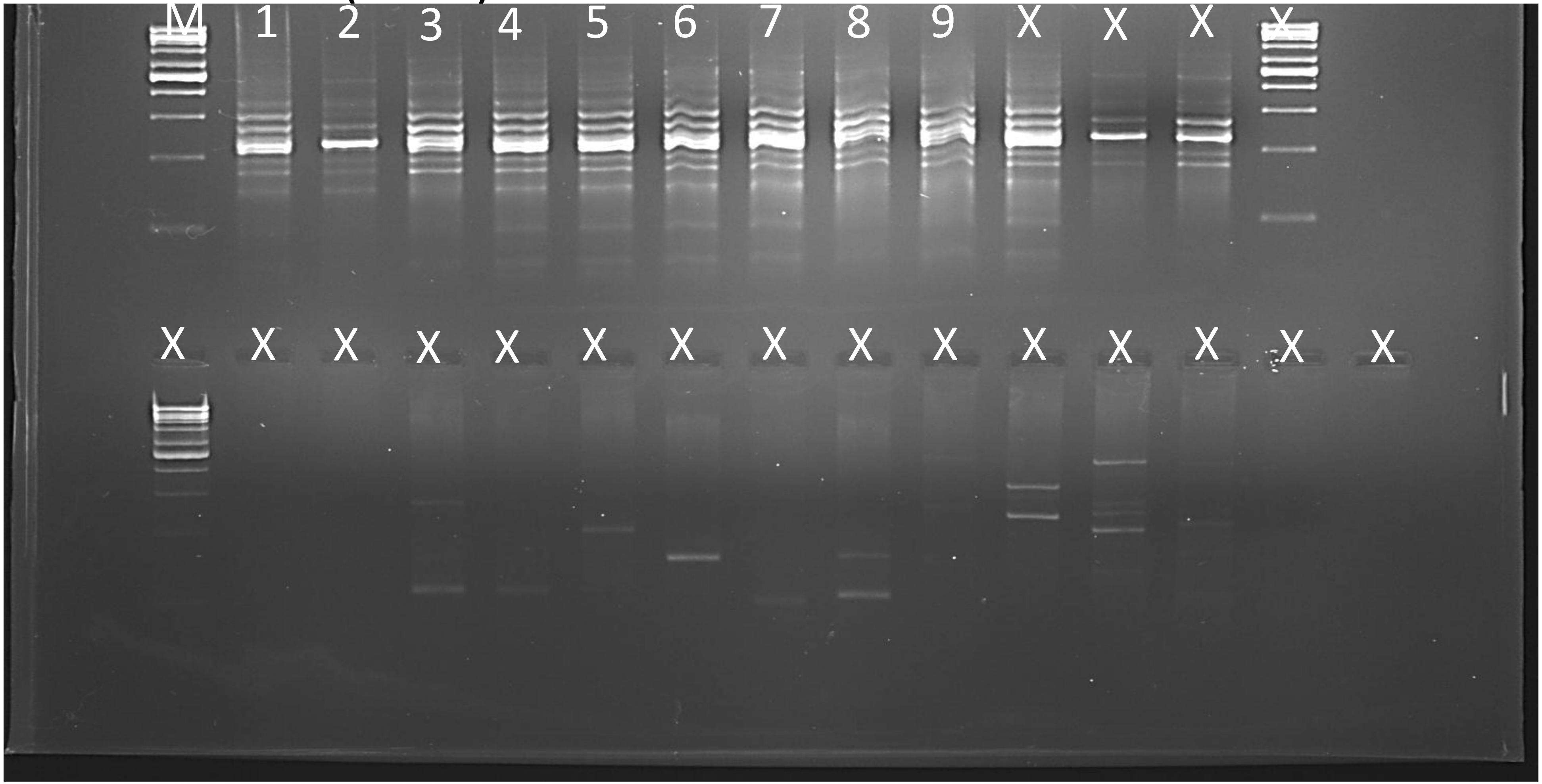

RAW IMAGES- RAPD (OPF-02)

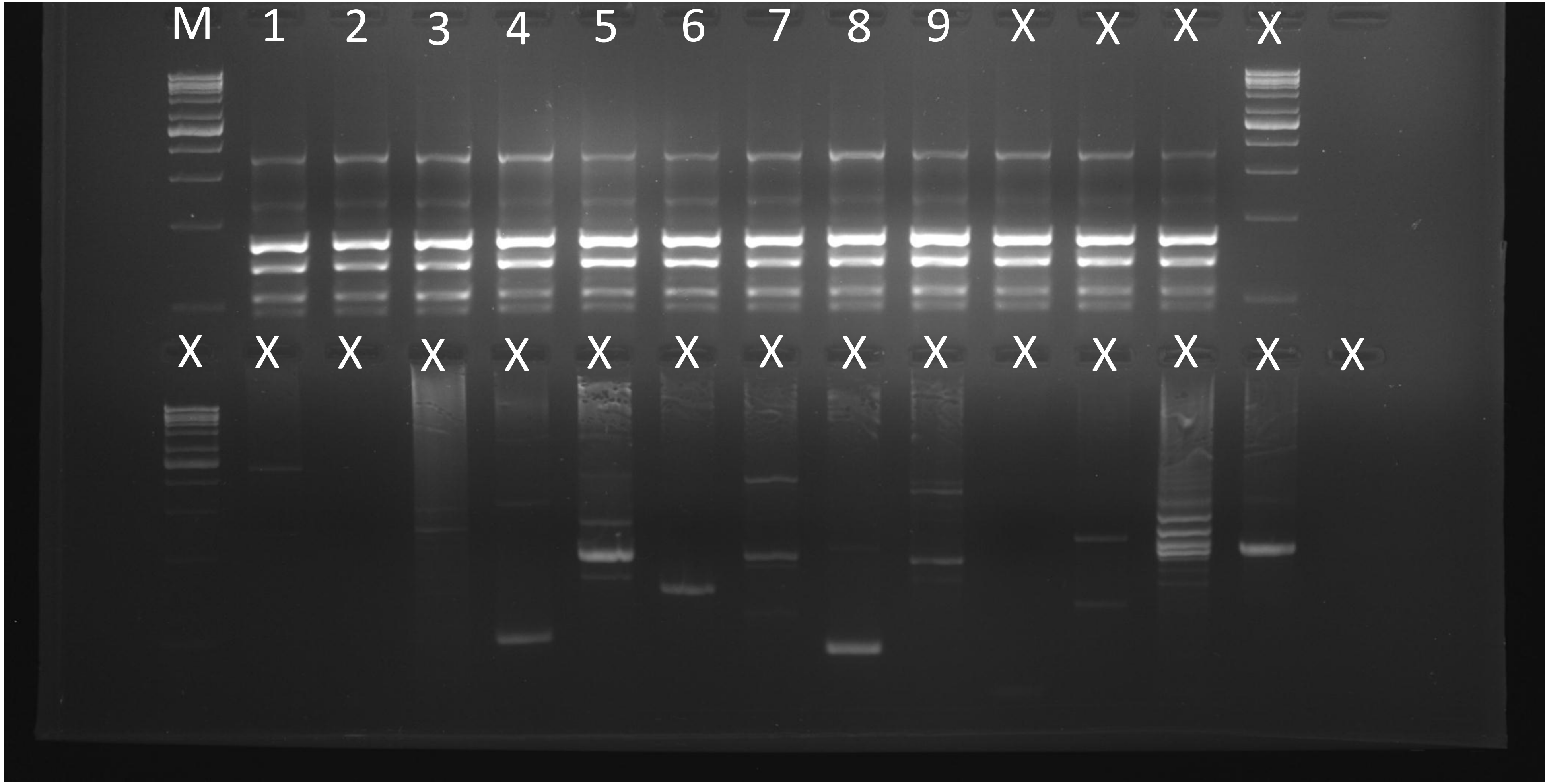

RAW IMAGES- RAPD (OPU-05)

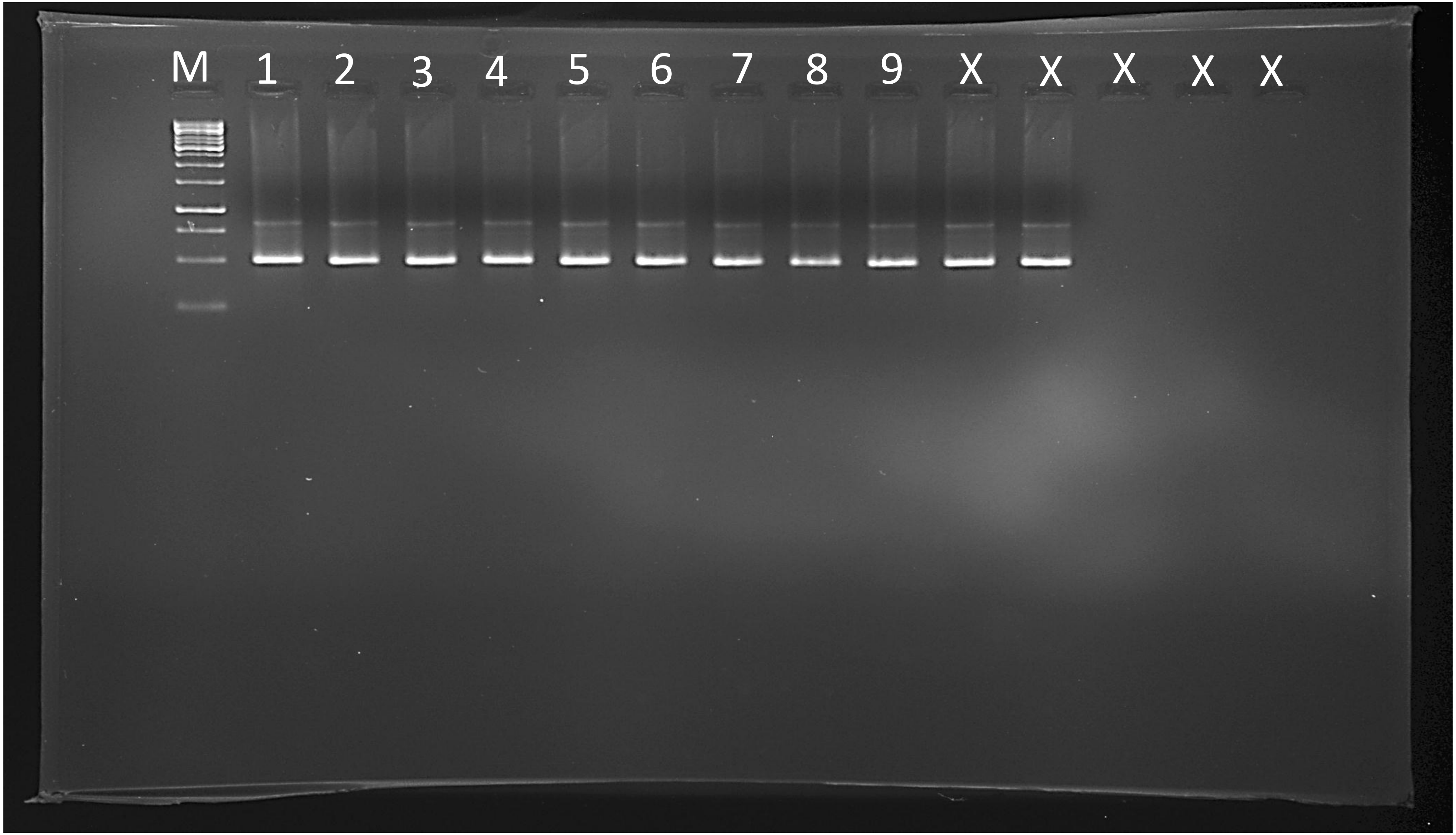

RAW IMAGES- RAPD (OPU-20)

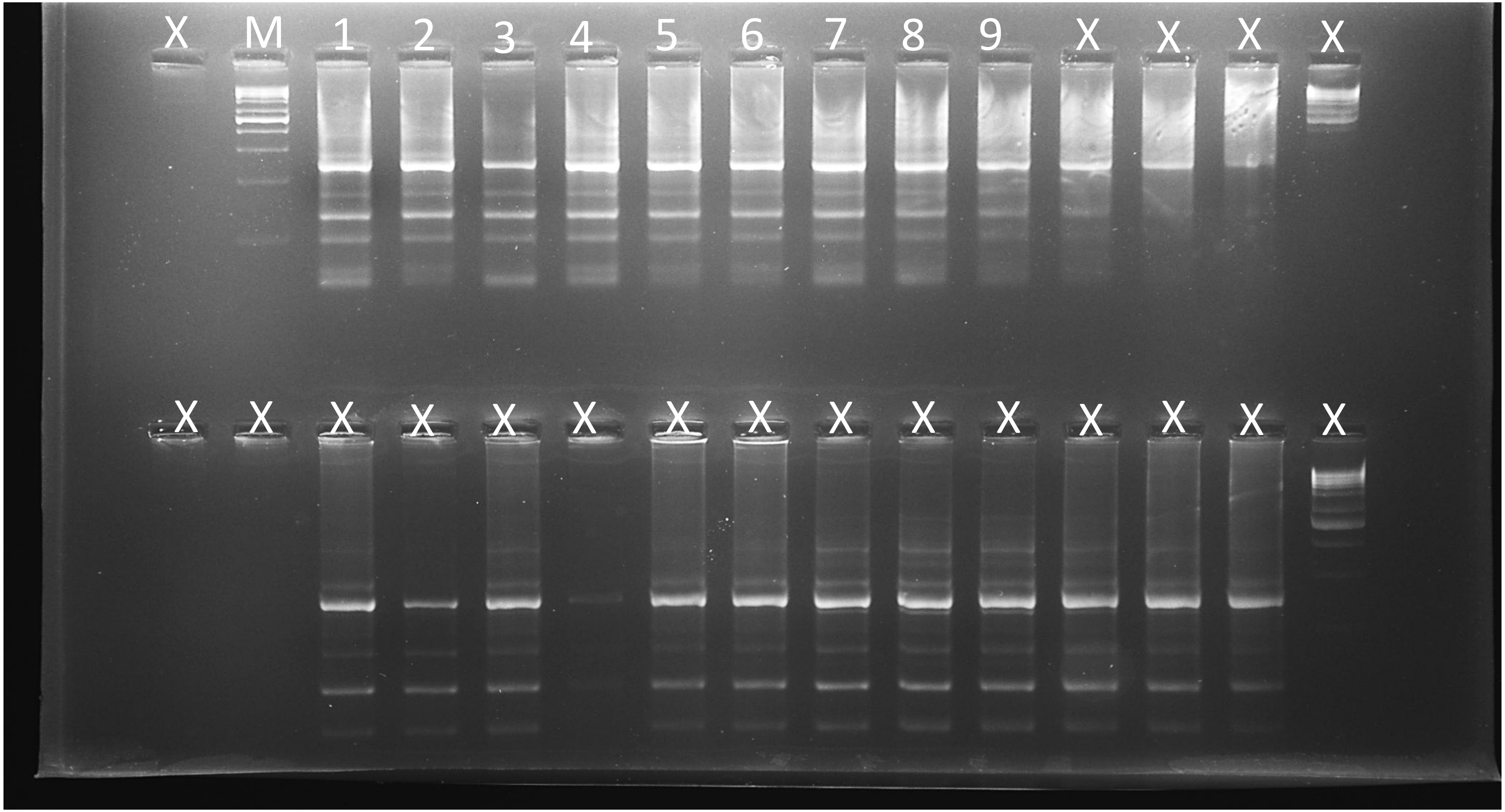

Supplement: S1 Raw images — (PDF) [file pone.0246971.s003.pdf]
