# Supplementary material for: High efficient de novo root-to-shoot organogenesis in Citrus jambhiri Lush.: Gene expression, genetic stability and virus indexing
Source: PLoS One. 2021 Feb 19;16(2):e0246971. doi: 10.1371/journal.pone.0246971 (PMC7894961; doi:10.1371/journal.pone.0246971)

RAW IMAGES-ISSR (UBC-807)

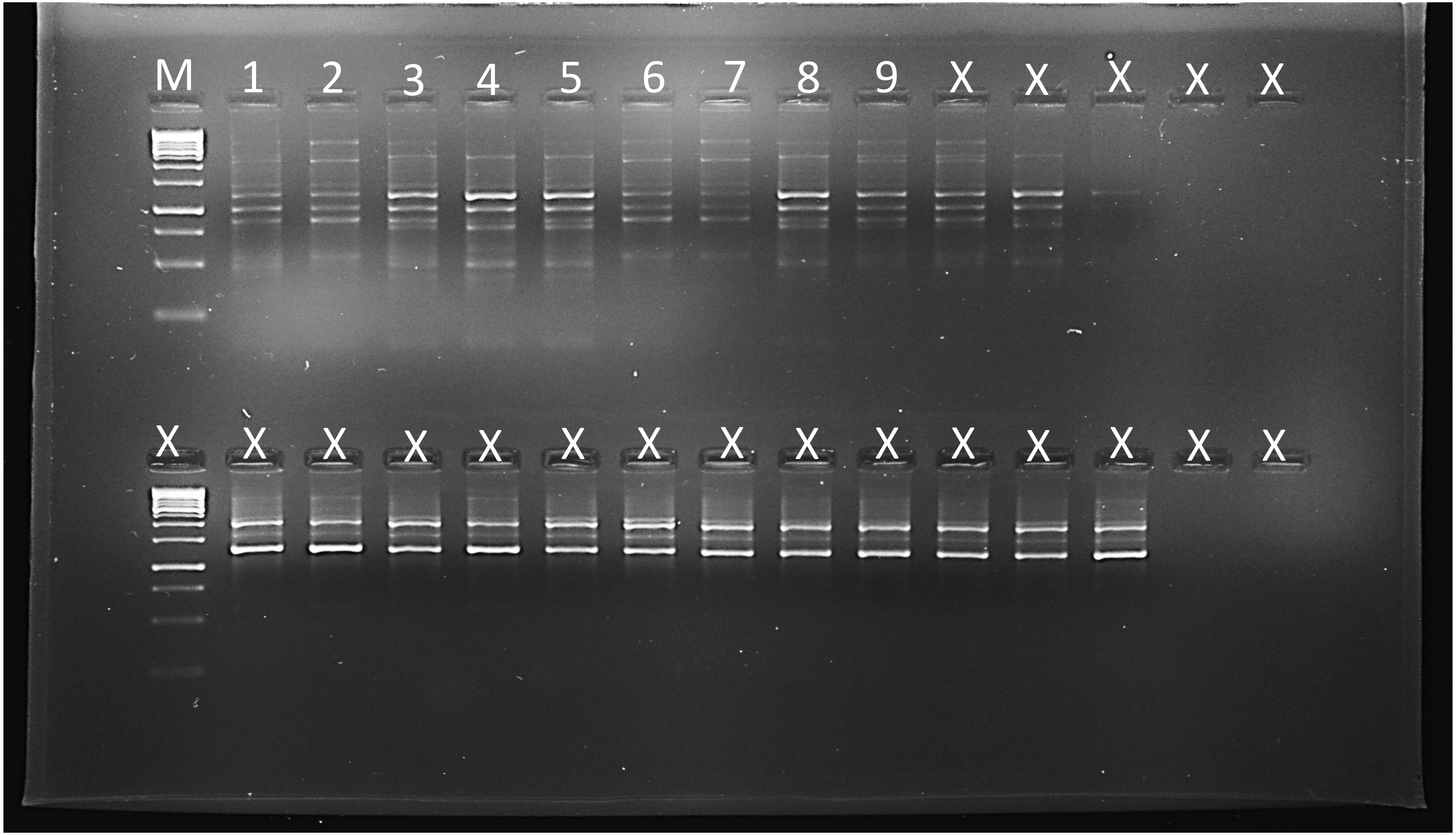

RAW IMAGES- ISSR (UBC-810)

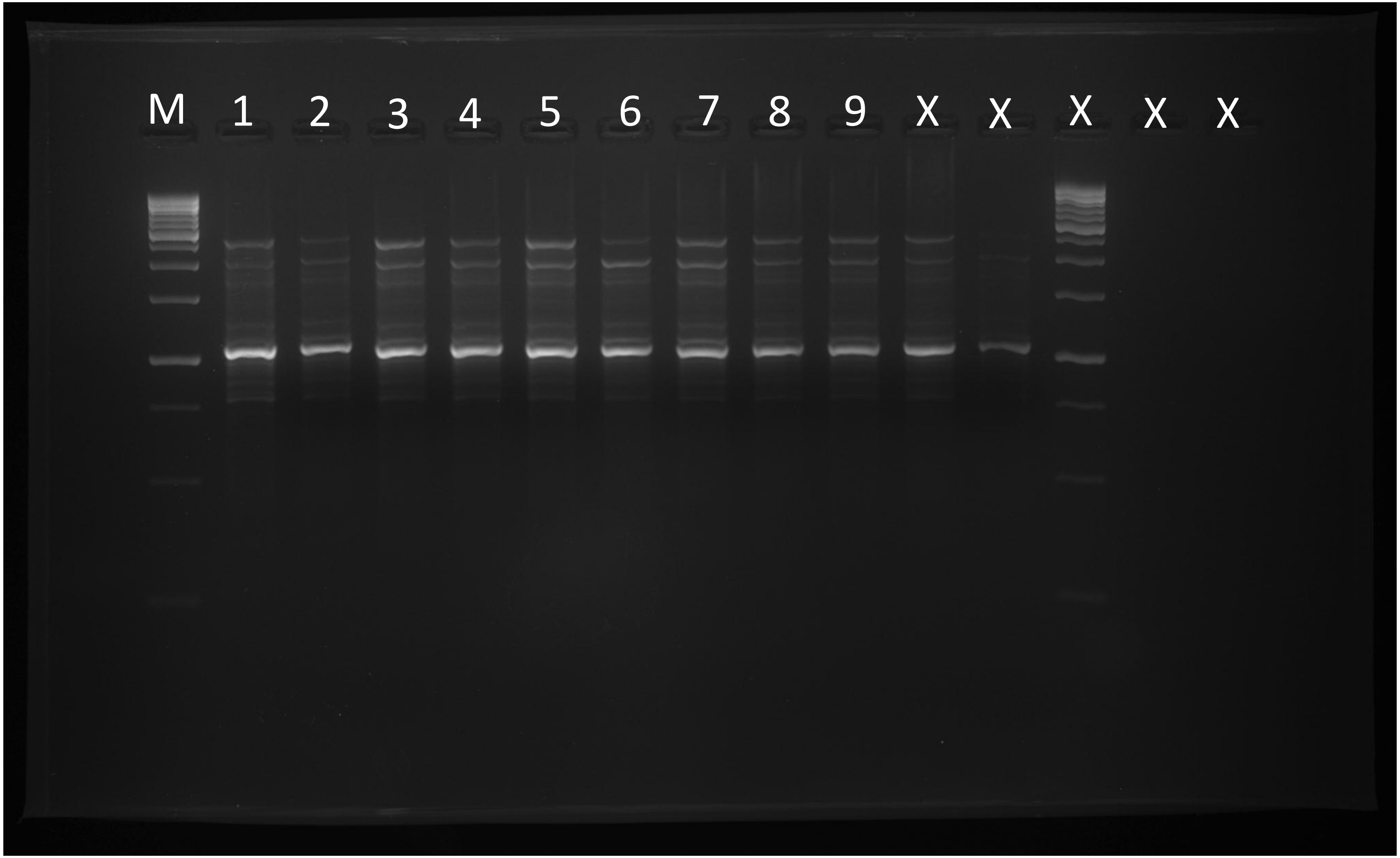

RAW IMAGES- ISSR (UBC-811)

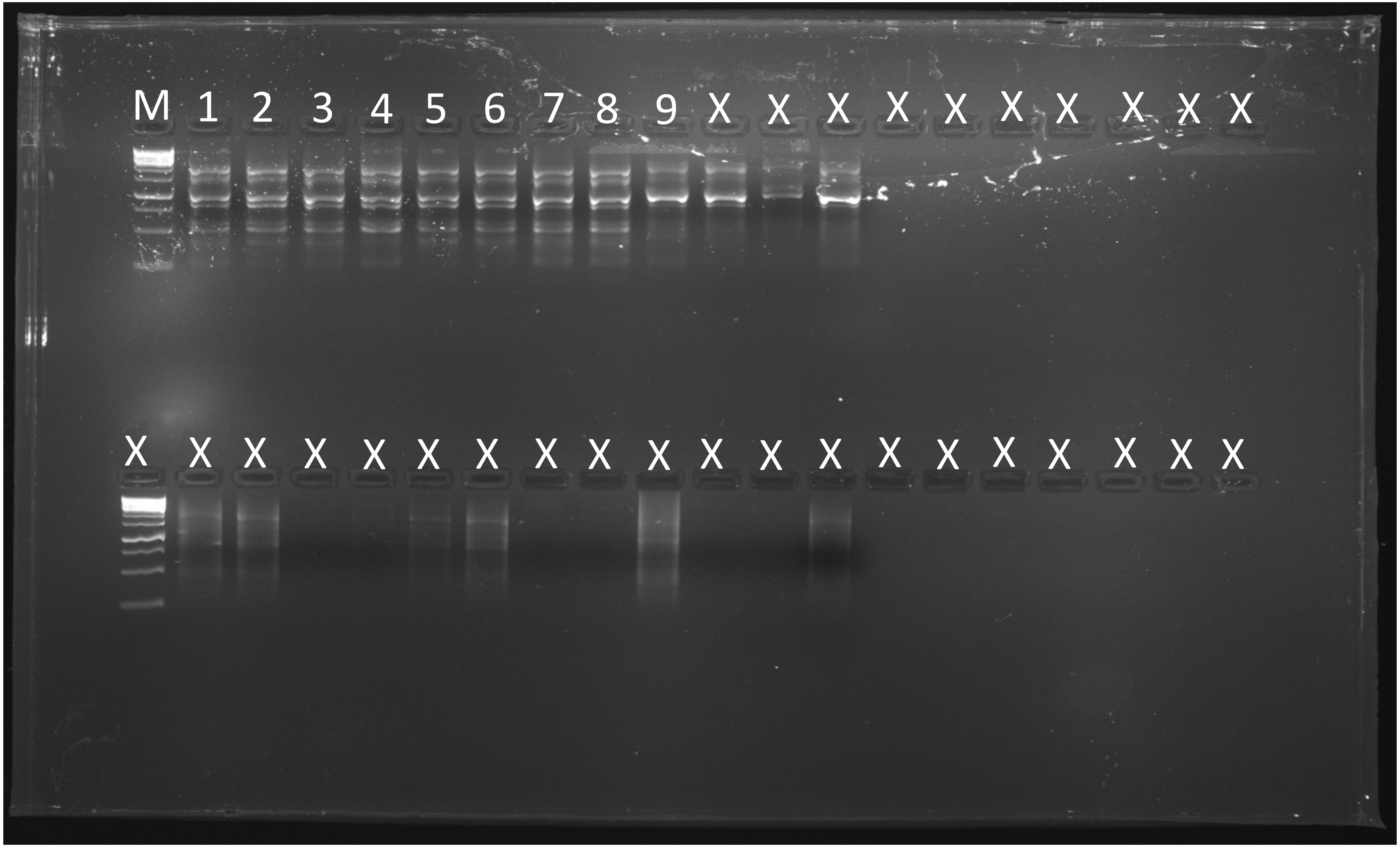

RAW IMAGES- ISSR (UBC-812)

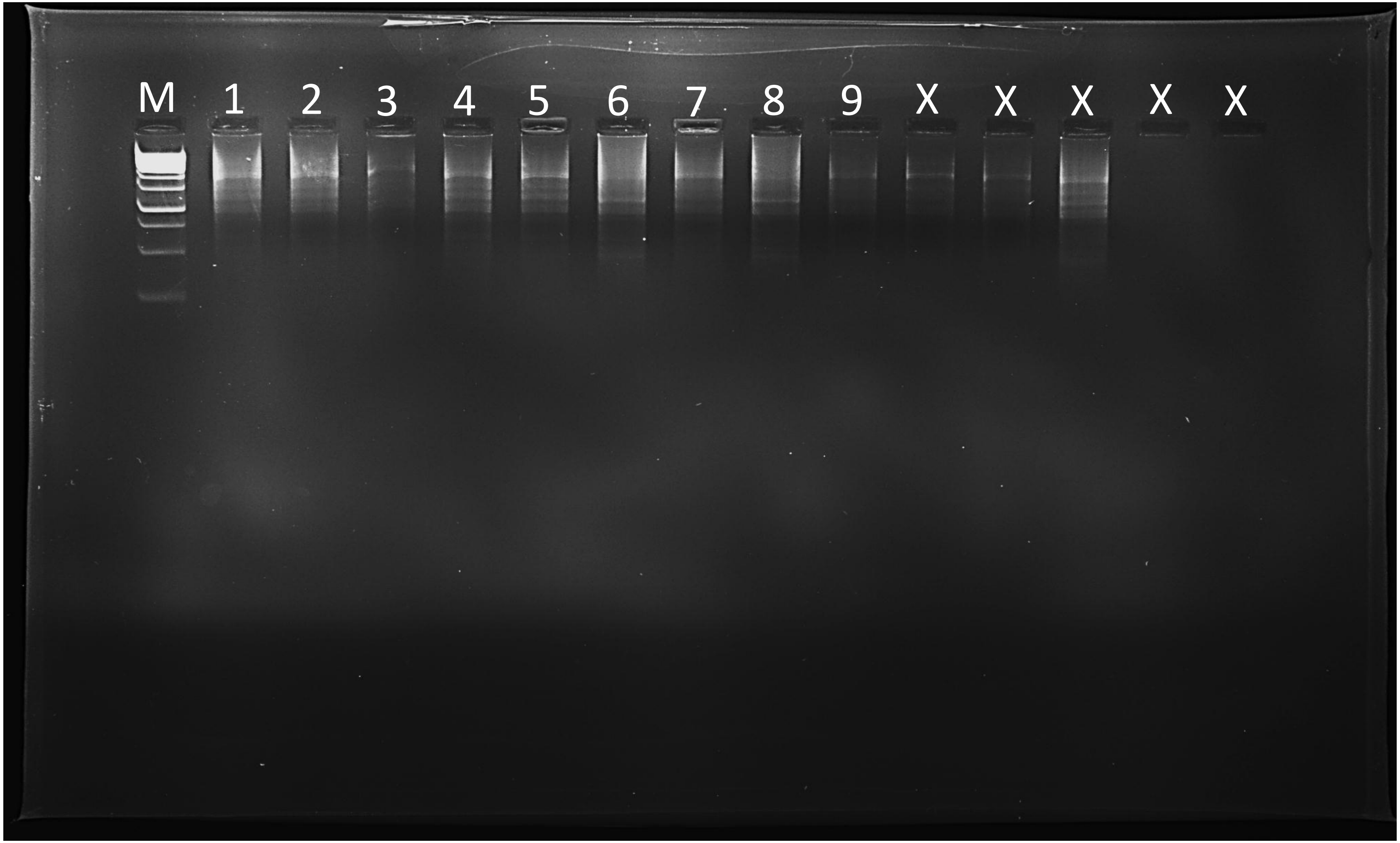

RAW IMAGES- ISSR (UBC-827)

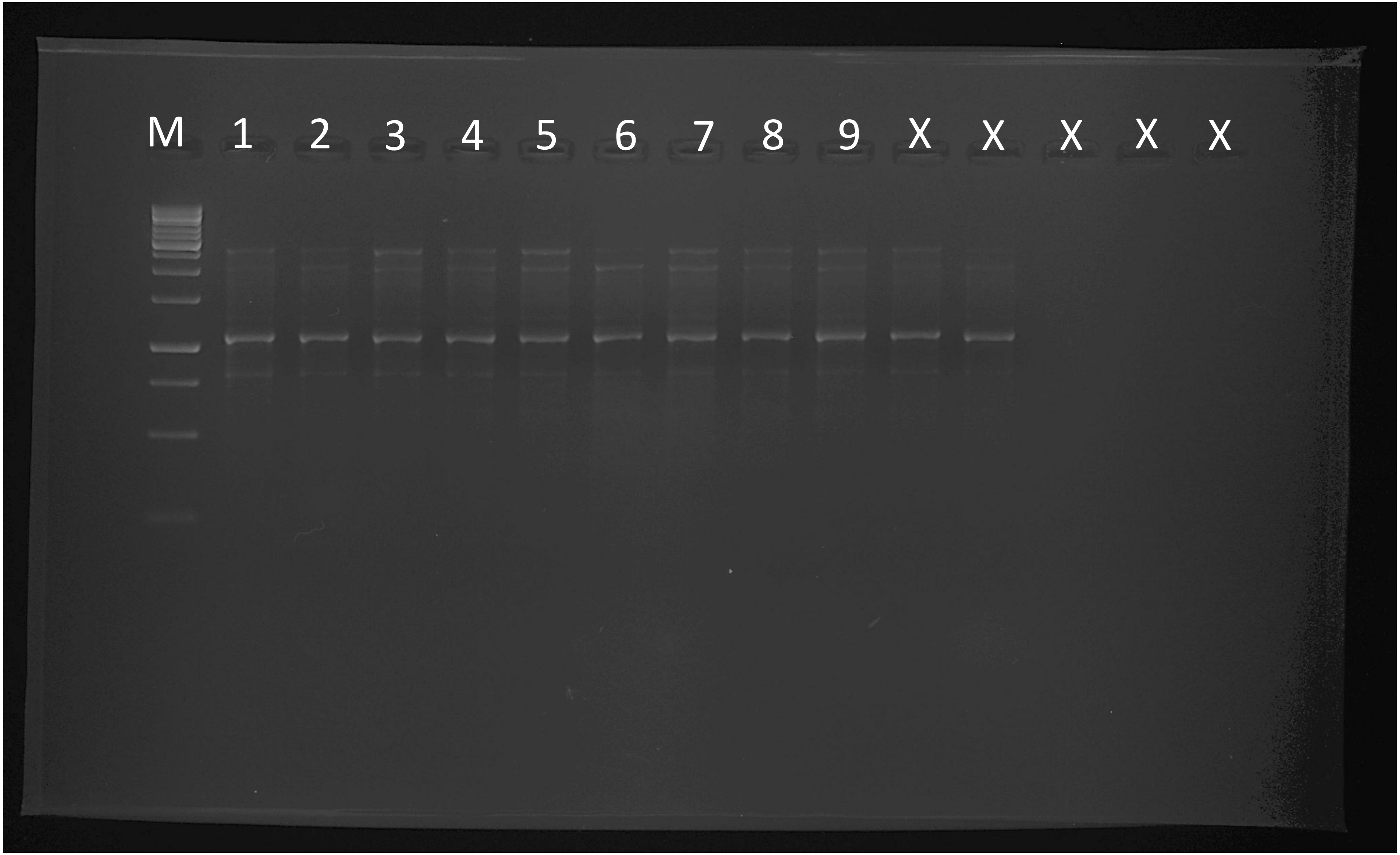

RAW IMAGES- ISSR (UBC-840)

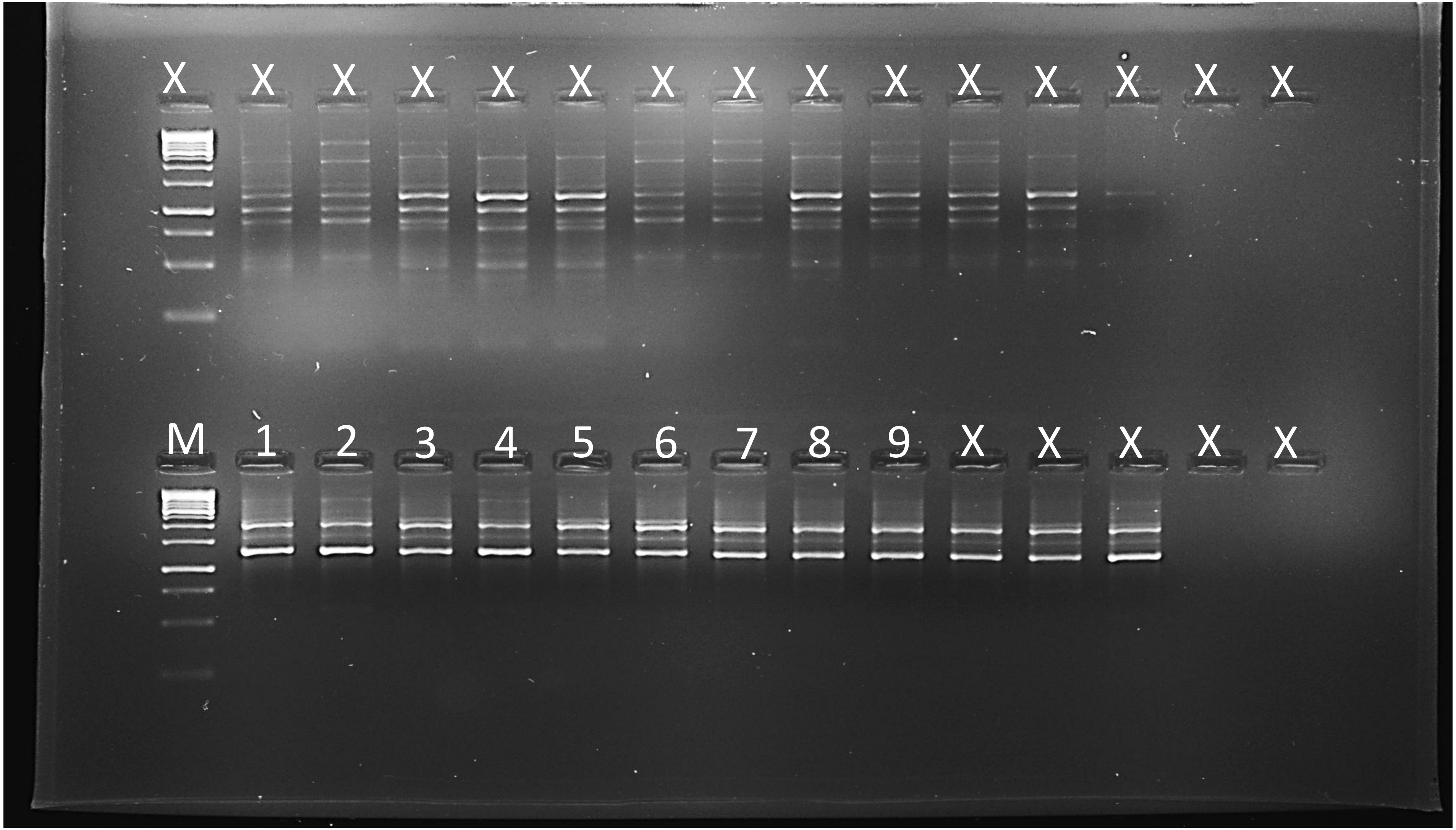

RAW IMAGES- ISSR (UBC-855)

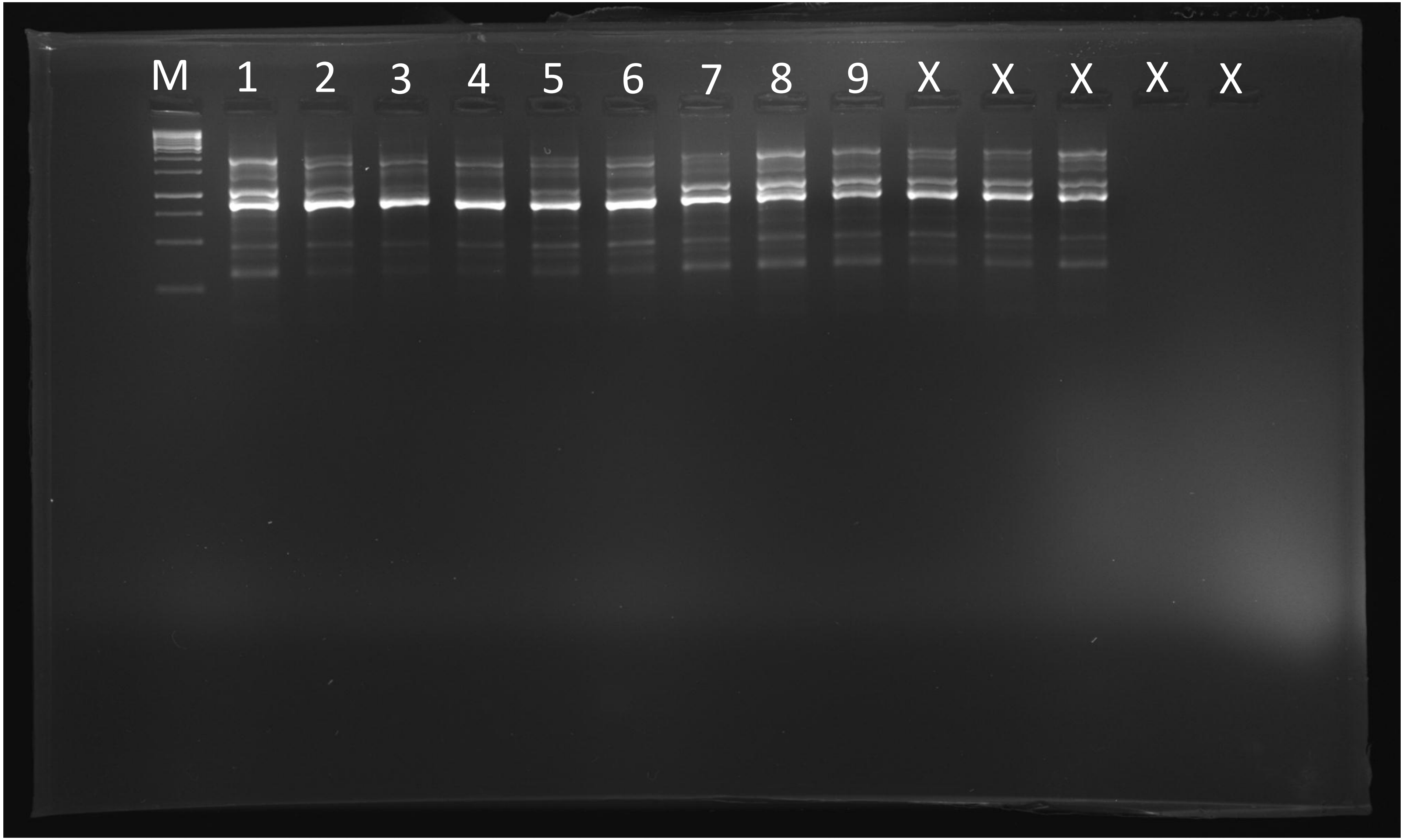

RAW IMAGES- ISSR (UBC-880)

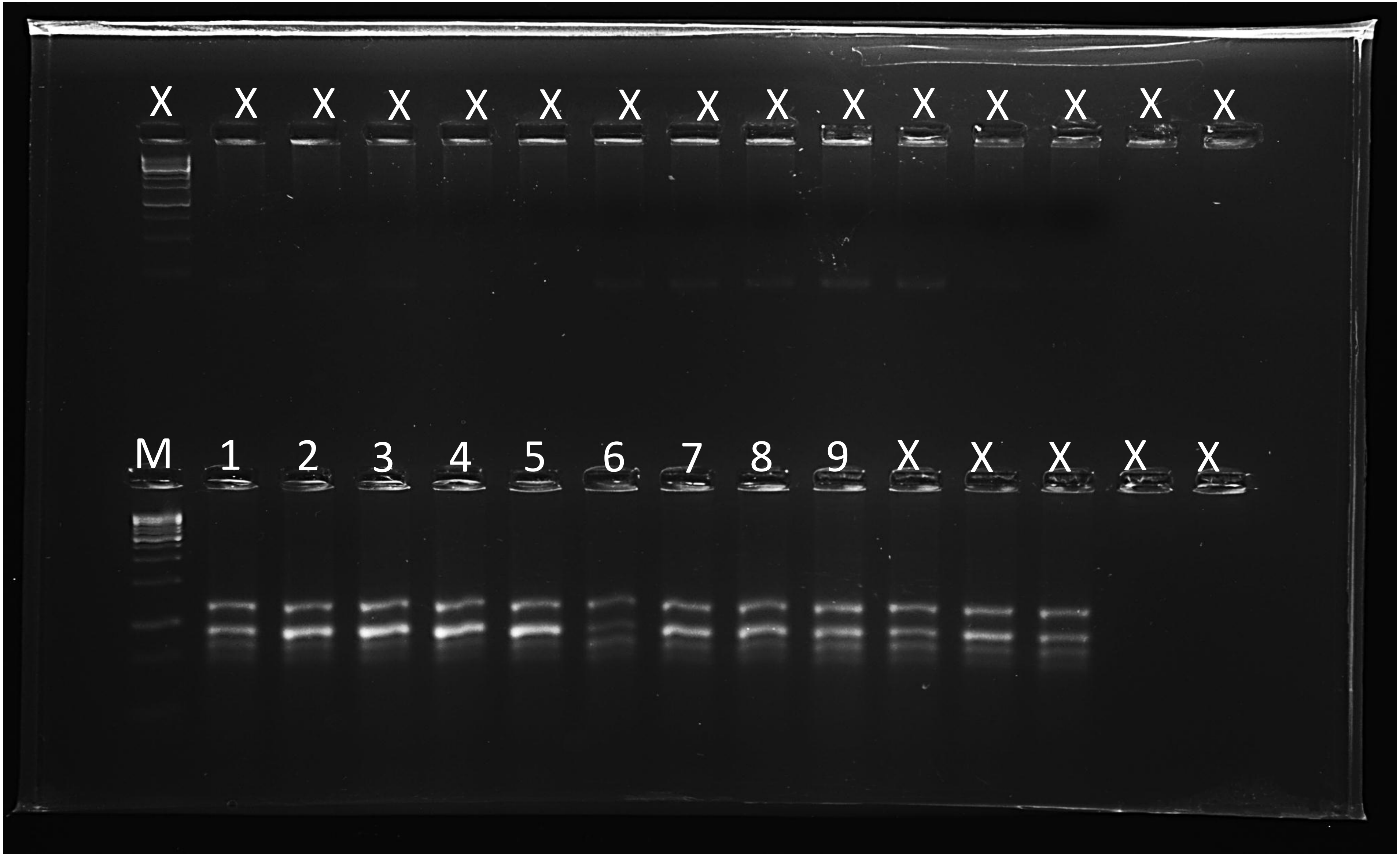

Supplement: S2 Raw images — (PDF) [file pone.0246971.s004.pdf]
