# Supplementary material for: High efficient de novo root-to-shoot organogenesis in Citrus jambhiri Lush.: Gene expression, genetic stability and virus indexing
Source: PLoS One. 2021 Feb 19;16(2):e0246971. doi: 10.1371/journal.pone.0246971 (PMC7894961; doi:10.1371/journal.pone.0246971)
Supplement: S1 Table — (DOCX) [file pone.0246971.s005.docx]

**S1 Table. List of quantitative real-time PCR (qRT-PCR) primers used for gene expression analyses of *Citrus jambhiri* Lush. regenerants**

| **Sl. Nos.** | **Primer Name** | **Primer Sequence** |
| --- | --- | --- |
| 1 | *GRF1* | 5’ CTTGGTTATTCGGGCAACAC 3’ |
|  |  | 5’ CCTTCCACAGGCTTTCTTGA 3’ |
| 2 | *GRF5* | 5’ CGAAATCCAGCAACACCTC 3’ |
|  |  | 5’ TCGGAGAGACTGACCTTCCT 3’ |
| 3 | *GA2OX1* | 5’ CTGGCCTCCAAATCCAACT 3’ |
|  |  | 5’ GTCATTACCTGCAAGGCATC 3’ |
| 4 | *KO1* | 5’ CAAGACTGGCGCTTCTACTA 3’ |
|  |  | 5’ TGGGTGGAAATGCTCAGAAGC 3’ |
| 5 | *PIN1* | 5’ GTTGATGGACCTGGAAGTGC 3’ |
|  |  | 5’ GCTAATGTGACGATGCCAAG 3’ |
| 6 | *PIN5* | 5’ CGTCAACACTAAAGCCACTGA 3’ |
|  |  | 5’ GGTCTGATGGAAGGAAACCA 3’ |
| 7 | *ARF1* | 5’ GGGCTGTTGATTTGACACG 3’ |
|  |  | 5’ CCACTTGCCACTTCTTGGTT 3’ |
| 8 | *ARF8* | 5’ CTCCCAACAAACAGCCAAC 3’ |
|  |  | 5’ ATCCCTCGCAATCAGTTCTT 3’ |
| 9 | *IAA4* | 5’ TGGGGCGCCATATCTAAGGA 3’ |
|  |  | 5’ AGCATCCAGTCGCCATCTTT 3’ |
| 10 | *GH3* | 5’ AGTTATGGCTGCGTCTGCTT 3’ |
|  |  | 5’ GGCAGTACATTTGGTGCTGA 3’ |
| 11 | *ACT 2* | 5’CCCTTCCTCATGCCATTCTTC 3’ |
|  |  | 5’ CGGCTGTGGTGGTAAACATGT 3’ |

DOI 10.17605/OSF.IO/JEG4K
