# Supplementary material for: High efficient de novo root-to-shoot organogenesis in Citrus jambhiri Lush.: Gene expression, genetic stability and virus indexing
Source: PLoS One. 2021 Feb 19;16(2):e0246971. doi: 10.1371/journal.pone.0246971 (PMC7894961; doi:10.1371/journal.pone.0246971)
Supplement: S2 Table — (DOCX) [file pone.0246971.s006.docx]

**S2 Table. List of RAPD and ISSR primers used for genetic fidelity studies of *Citrus jambhiri* Lush. regenerants**

| **Sl. Nos.** | **Primer** | **Sequence (5’-3’)** |
| --- | --- | --- |
| **RAPD Primers** | | |
| 1 | OPA 09 | 5’ GGGTAACGCC 3’ |
| 2 | OPC 01 | 5’ TTCGAGCCAG 3’ |
| 3 | OPC 08 | 5’ TGGACCGGTG 3’ |
| 4 | OPC 12 | 5’ TGTCATCCCC 3’ |
| 5 | OPD 02 | 5’ GGACCCAACC 3’ |
| 6 | OPF 02 | 5’ GAGGATCCCT 3’ |
| 7 | OPU 05 | 5’ TTGGCGGCCT 3’ |
| 8 | OPU 20 | 5’ ACAGCCCCCA 3’ |
| **ISSR Markers** | | |
| 9 | UBC-807 | (AG)8T |
| 10 | UBC-810 | (GA)8T |
| 11 | UBC-811 | (GA)8C |
| 12 | UBC-812 | (GA)8A |
| 13 | UBC-827 | (AC)8G |
| 14 | UBC-840 | (GA)8YT |
| 15 | UBC-855 | (AC)8YT |
| 16 | UBC-880 | (GGGTG)3 |

DOI 10.17605/OSF.IO/KVUX9
