# Supplementary material for: High efficient de novo root-to-shoot organogenesis in Citrus jambhiri Lush.: Gene expression, genetic stability and virus indexing
Source: PLoS One. 2021 Feb 19;16(2):e0246971. doi: 10.1371/journal.pone.0246971 (PMC7894961; doi:10.1371/journal.pone.0246971)
Supplement: Attachment — Submitted filename: Response to Reviewers.docx [file pone.0246971.s007.docx]

**Response to the Reviewers/Editors**

| **Sl** | **Reviewers/Editors comments** |  | **Author’s response** |
| --- | --- | --- | --- |
|  | **Reviewer#1** | : |  |
|  | The manuscript entitled “High efficient de novo root-to-shoot organogenesis in Citrus *jambhiri* Lush.: gene expression, genetic stability, and virus indexing” is nicely planned and executed. Though it can be considered for possible publication in Plos One, the current version still needs improvement. Please address the below-mentioned comments for further improvement. | | |
| 1 | Line 28, 45, please mention the common name on the first appearance in the abstract and the main text | : | Revised as suggested [Line 30, 50] |
| 2 | Line 37, please follow the standard unit style throughout the text, i.e., mg L-1 | : | Revised as suggested |
| 3 | Line 86, define RAPD and ISSR. Why authors choose RAPD markers along with ISSR? Although the experiment is well planned and executed, however, I got some reservations concerning RAPD markers that are known to have low reproducibility and thus can impact the reliability of results. If the experiment is replicated to ensure the PCR results were reproducible, then there are no issues with the accuracy of results given in this manuscript. | : | Revised as suggested (Line 92-93)  In this study, we have assessed the genetic fidelity using RAPD markers (most commonly used DNA marker in Citrus spp.) and further confirmed with the ISSR markers.  The advantage of ISSR over RAPD has been discussed in the ‘Discussion’ section (Line 409-410).  The experiment was replicated twice to ensure the reproducibility. |
| 4 | Line 92, define GA. | **:** | Revised as suggested (gibberellin) [Line 99] |
| 5 | Line 219-222, please mention which statistical tool/software was used for the data analysis | : | The statistical analyses were performed using XLSTAT statistical software (XLSTAT Premium 2020.2.1, Adinsoft, NY) [Line 236-237]. |
| 6 | Please provide a sharper image for fig 6. | : | The sharper image of fig. 6 has been furnished |
| 7 | In section “Assessment of genetic stability”, please provide the complete data sets in terms of remaining gel images or arrange all the generated data in a table (suppl. table/figures). Currently, there are only two gel images for two primers only. How about the results of other markers? Therefore, please provide us with the other data as a suppl. material. | : | The details of the primer sequences, amplified bands and band size have been presented in Table-3.  The representative RAPD (OPU05) and ISSR (UBC810) gel images have been presented in Fig. 7.  All the remaining gel images have been provided as supplementary figure. 1 and 2 (S1 and S2 Figs.) |
| 8 | Please be consistent with analyse or anlyze. Also, check the entire text for grammar and spacing errors. The English language needs improvement. | : | The term ‘analyse’ was used throughout the text.  The grammar, English language and spacing errors were re-checked using grammarly software. |
|  | **Reviewer#2** | : |  |
| 1 | In this paper the authors showed the “High efficient de novo root-to-shoot organogenesis in Citrus jambhiri Lush.: gene expression, genetic stability and virus indexing”. The authors have studied some parameters related to phenomenon of root-to-shoot organogenesis. This is a well written article and standard for such a high-ranking journal. Overall, it is a good presented paper. Language quality is need be improved. I could recommend it for publication | **:** | The language has been improved using grammarly software. |
|  | **Reviewer#3** | : |  |
| 1 | Please add the novelty and aims to ABSTRACT. | : | The novelty and aim (Line 43-46) of the study has been incorporated in the ABSTRACT as suggested. |
| 2 | INTRODUCTION is so long, please revise it | : | Introduction section has been revised as suggested. |
| 3 | All figures related to gene expression are not professionally drawn, so I suggest to be redrawn them with a suitable software | : | The figures related to gene expression have been redrawn to violin plot using origin software. |
|  | **Academic Editor** | **:** |  |
| 1 | A rebuttal letter that responds to each point raised by the academic editor and reviewer(s). You should upload this letter as a separate file labeled 'Response to Reviewers' | : | The file containing the ‘Response to reviewers’ has been uploaded |
| 2 | A marked-up copy of your manuscript that highlights changes made to the original version. You should upload this as a separate file labeled 'Revised Manuscript with Track Changes' | : | The marked copy 'Revised Manuscript with Track Changes' has been uploaded |
| 3 | An unmarked version of your revised paper without tracked changes. You should upload this as a separate file labeled 'Manuscript' | : | The unmarked copy ‘Manuscript' has been uploaded |
| 4 | If you would like to make changes to your financial disclosure, please include your updated statement in your cover letter. | : | The cover letter has been uploaded |
| 5 | Guidelines for resubmitting your figure files are available below the reviewer comments at the end of this letter. | : | The figure files are uploaded adhering to the guidelines |
| 6 | If applicable, we recommend that you deposit your laboratory protocols in protocols.io to enhance the reproducibility of your results. Protocols.io assigns your protocol its own identifier (DOI) so that it can be cited independently in the future. For instructions see: <http://journals.plos.org/plosone/s/submission-guidelines#loc-laboratory-protocols> | : | The laboratory protocol has been deposited in protocols.io as suggested.  ([dx.doi.org/10.17504/protocols.io.bq4emyte](https://dx.doi.org/10.17504/protocols.io.bq4emyte)) |
| 7 | Please ensure that your manuscript meets PLOS ONE's style requirements, including those for file naming. The PLOS ONE style templates can be found at https://journals.plos.org/plosone/s/file?id=wjVg/PLOSOne_formatting_sample_main_body.pdf and <https://journals.plos.org/plosone/s/file?id=ba62/PLOSOne_formatting_sample_title_authors_affiliations.pdf> | : | The manuscript has been revised adhering to the PLOS ONE style templates |
| 8 | In your Methods section, please provide additional location information, including geographic coordinates for the data set if available. | : | Location information of the collected samples has been provided (Line 125; [25°14′ N and 94°16′ E](https://geohack.toolforge.org/geohack.php?pagename=Kachai&params=25_14_29_N_94_16_22_E_type:city(2107)_region:IN-MN) and 1662 m) |
| 9 | In your Methods section, please provide additional information regarding the permits you obtained for the work. Please ensure you have included the full name of the authority that approved the field site access and, if no permits were required, a brief statement explaining why. | : | No permit is required for collection of the plant material for the present study. |
| 10 | We note that you have indicated that data from this study are available upon request. PLOS only allows data to be available upon request if there are legal or ethical restrictions on sharing data publicly. For more information on unacceptable data access restrictions, please see http://journals.plos.org/plosone/s/data-availability#loc-unacceptable-data-access-restrictions. | : | We have no legal or ethical restrictions on sharing data publicly. |
| 11 | In your revised cover letter, please address the following prompts: a) If there are ethical or legal restrictions on sharing a de-identified data set, please explain them in detail (e.g., data contain potentially sensitive information, data are owned by a third-party organization, etc.) and who has imposed them (e.g., an ethics committee). Please also provide contact information for a data access committee, ethics committee, or other institutional body to which data requests may be sent.  b) If there are no restrictions, please upload the minimal anonymized data set necessary to replicate your study findings as either Supporting Information files or to a stable, public repository and provide us with the relevant URLs, DOIs, or accession numbers. For a list of acceptable repositories, please see <http://journals.plos.org/plosone/s/data-availability#loc-recommended-repositories>.  We will update your Data Availability statement on your behalf to reflect the information you provide. | : | As declared above, we have no legal or ethical restrictions on sharing a de-identified data set publicly.  The minimal anonymized data set has been uploaded in the suggested public repository  URLs: https://osf.io/myprojects/  DOIs:  Table 1: DOI 10.17605/OSF.IO/F9U35  Table 2: DOI 10.17605/OSF.IO/P7SFZ  Table 3: DOI 10.17605/OSF.IO/RXQHS  S1Table: DOI 10.17605/OSF.IO/JEG4K  S2 Table: DOI 10.17605/OSF.IO/KVUX9 |
| 12 | PLOS ONE now requires that authors provide the original uncropped and unadjusted images underlying all blot or gel results reported in a submission’s figures or Supporting Information files. This policy and the journal’s other requirements for blot/gel reporting and figure preparation are described in detail at https://journals.plos.org/plosone/s/figures#loc-blot-and-gel-reporting-requirements and https://journals.plos.org/plosone/s/figures#loc-preparing-figures-from-image-files. When you submit your revised manuscript, please ensure that your figures adhere fully to these guidelines and provide the original underlying images for all blot or gel data reported in your submission. See the following link for instructions on providing the original image data: <https://journals.plos.org/plosone/s/figures#loc-original-images-for-blots-and-gels>.  In your cover letter, please note whether your blot/gel image data are in Supporting Information or posted at a public data repository, provide the repository URL if relevant, and provide specific details as to which raw blot/gel images, if any, are not available. Email us at plosone@plos.org if you have any questions. | : | The figures are arranged adhering to the journal guidelines and the original uncropped/unadjusted images have been presented in the manuscript.  The original images/figures have been posted at the public data repository vide DOIs;  Fig. 1: DOI 10.17605/OSF.IO/CFT75  Fig. 2: DOI 10.17605/OSF.IO/FR7X5  Fig. 3: DOI 10.17605/OSF.IO/XGUFT  Fig. 4: DOI 10.17605/OSF.IO/C27QB  Fig. 5: DOI 10.17605/OSF.IO/GP6AH  Fig. 6: DOI 10.17605/OSF.IO/YDNKU  Fig. 7: DOI 10.17605/OSF.IO/9YC6V  Fig. 8: DOI 10.17605/OSF.IO/R67JZ  S1Fig: DOI 10.17605/OSF.IO/XWT8B  S2Fig: DOI 10.17605/OSF.IO/JBZUX |
| 13 | Please include captions for your Supporting Information files at the end of your manuscript, and update any in-text citations to match accordingly. Please see our Supporting Information guidelines for more information: [http://journals.plos.org/plosone/s/supporting-information](about:blank) | : | The captions for the supporting documents have been included at the end of the manuscript. |
| 14 | If reviewer comments were submitted as an attachment file, they will be attached to this email and accessible via the submission site. Please log into your account, locate the manuscript record, and check for the action link "View Attachments". If this link does not appear, there are no attachment files |  | There is no attachment files appeared in ‘View Attachments’ |
| 15 | While revising your submission, please upload your figure files to the Preflight Analysis and Conversion Engine (PACE) digital diagnostic tool, <https://pacev2.apexcovantage.com/>. PACE helps ensure that figures meet PLOS requirements. To use PACE, you must first register as a user. Registration is free. Then, login and navigate to the UPLOAD tab, where you will find detailed instructions on how to use the tool. If you encounter any issues or have any questions when using PACE, please email PLOS at [figures@plos.org](mailto:figures@plos.org). Please note that Supporting Information files do not need this step. |  | The figure files have been uploaded to the PACE digital diagnostic tool at <https://pacev2.apexcovantage.com> |
